# Solitary adrenal metastasis presenting 15 years after breast cancer: a case report

**DOI:** 10.1097/MS9.0000000000004138

**Published:** 2025-10-28

**Authors:** Salsabil Haque, Jamil Alghanem, Suven Shankar

**Affiliations:** a College of Medicine, Alfaisal University, Riyadh, KSA; b Department of Surgery, SIH Cancer Institute, Southern Illinois Healthcare, Carbondale, IL, USA

**Keywords:** adrenal metastasis, breast neoplasms, case report, late-onset metastases

## Abstract

**Introduction and importance:**

Adrenal metastasis from breast cancer is exceptionally rare and typically arises within a few years of the primary diagnosis. We report a unique case of solitary adrenal metastasis presenting 15 years after breast carcinoma, with hypercalcemia as the only symptom.

**Presentation of Case:**

A 67-year-old woman with a history of estrogen receptor/progesterone receptor positive breast adenocarcinoma diagnosed 15 years earlier presented with hypercalcemia. Imaging revealed a solitary left adrenal mass with no evidence of other metastases. She underwent an open left adrenalectomy. Histopathology confirmed metastatic breast carcinoma. Postoperatively, serum calcium normalized, and recovery was uneventful.

**Clinical Discussion:**

Solitary adrenal metastasis from breast cancer is rare, particularly after a decade-long latency. In carefully selected patients, surgical resection can provide symptom relief and may offer survival benefit.

**Conclusion:**

This case highlights that isolated adrenal metastasis can occur even after prolonged remission, with hypercalcemia as the sole presenting feature. It emphasizes the need for long-term surveillance in breast cancer survivors and the potential role of surgery in managing isolated adrenal metastases.

## Background and rationale

Several primary neoplasms metastasize to the adrenal glands, with certain malignancies showing a high propensity to do so. Lung cancer (39%), breast cancer (35%), renal cell carcinoma, melanoma, and colorectal cancer are the most common primary neoplasms to spread to the adrenal gland^[1]^. They predominantly occur through systemic hematogenous dissemination of primary malignancy^[2,3]^. Although breast cancer can involve the adrenal glands, such metastases are relatively uncommon^[4]^. More importantly, *isolated* adrenal metastasis from breast cancer, especially as a late-onset metachronous lesion, remains a rare clinical presentation^[4]^. Breast cancer metastases are generally associated with poor prognosis, with a 5-year survival rate of only around 10%^[5]^. Due to the aforementioned rarity of this entity, a standardized management plan is not yet established. The treatment of adrenal gland metastases typically involves a multidisciplinary approach, including surgical intervention, radiation therapy, and systemic therapies, depending on the extent of the disease and the primary cancer type. Here, we report a rare case of resection of adrenal gland metastases in a patient with advanced breast cancer diagnosed 15 years ago. This case report is reported in line with the SCARE checklist^[6]^.

**HIGHLIGHTS**

- First reported case of solitary adrenal metastasis from breast cancer 15 years postdiagnosis presenting with PTHrp-related hypercalcemia.
- Surgical adrenalectomy-enabled diagnosis and resolved symptoms, supporting its role in selected cases.
- Emphasizes need for long-term surveillance and biochemical assessment of adrenal masses in survivors.

## Presentation of case

**Figure 1.**
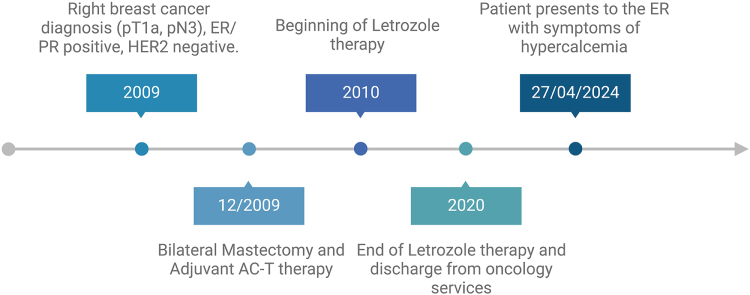
Timeline of clinical events. The patient was diagnosed with right breast cancer (pT1a, pN3), estrogen receptor (ER) and progesterone receptor (PR) positive, and human epidermal growth factor receptor 2 (HER2) negative. She underwent bilateral mastectomy and adjuvant AC-T chemotherapy (Adriamycin and Cyclophosphamide followed by Taxane), followed by 10 years of letrozole therapy. She was discharged from oncology care in 2020 and presented to the emergency room in April 2024 with symptomatic hypercalcemia. Created with Biorender.

Fig. 1 illustrates the timeline of clinical events of our patient. A 67-year-old woman was sent to the emergency department by her primary care physician on April 27, 2024 due to hypercalcemia (Ca = 13.5 mg/dL). On admission, she was vitally stable; however, her body mass index (BMI) was 38.7 kg/m^2^ (class II obesity). She has a significant medical history of hormone receptor-positive breast cancer, diagnosed in 2009, for which she underwent a bilateral mastectomy. Her treatment also included adjuvant Adriamycin and cyclophosphamide, followed by Taxol (AC-T) chemotherapy, radiation therapy, and a 10-year course of letrozole, after which she was cleared from oncology follow-up in 2020. Her past social history is significant for 17 pack-years of smoking, which she quit 16 years ago. Other comorbidities include congestive heart failure, hyperlipidemia, hypertension, and hypothyroidism. Family history of malignancy is positive in her maternal grandmother.

**Figure 2.**
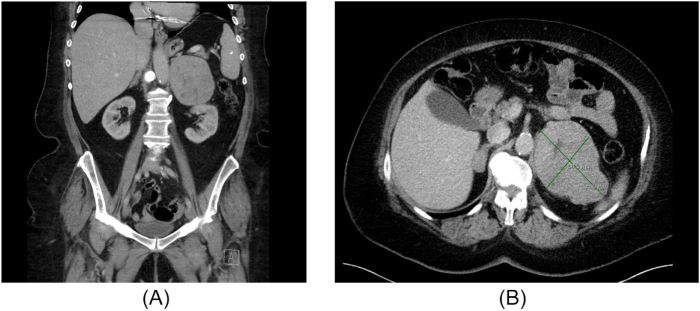
Figure A and B show axial and coronal CT scans, respectively. A slightly heterogenous mass of 8.4 cm by 11.2 cm is seen in the left adrenal gland.

Hypercalcemia has a wide set of differentials, including primary hyperparathyroidism, malignancy, bone metastases, and paraneoplastic syndromes. Hence, appropriate blood investigations were performed, which revealed a markedly elevated PTHrP level (251.0 pmol/L). All other values, namely— parathyroid hormone, cortisol, plasma and urine metanephrine, catecholamine, and dopamine levels were within the normal range. A computed topography (CT) scan of the abdomen, performed as part of her evaluation revealed a lobular left adrenal mass, measuring 11.3 × 8.2 cm (Fig. 2a-b). Based on the size and symptoms of hormone production, she was subsequently referred to surgical oncology.

The patient underwent an open left adrenalectomy in June 2024 under general endotracheal tube anesthesia. The mass measured 15 cm × 14 cm × 10 cm and weighed 520.7 g. Postoperative PTHrp levels have dropped to 7.9 pmol/L and calcium levels have normalized.

**Figure 3.**
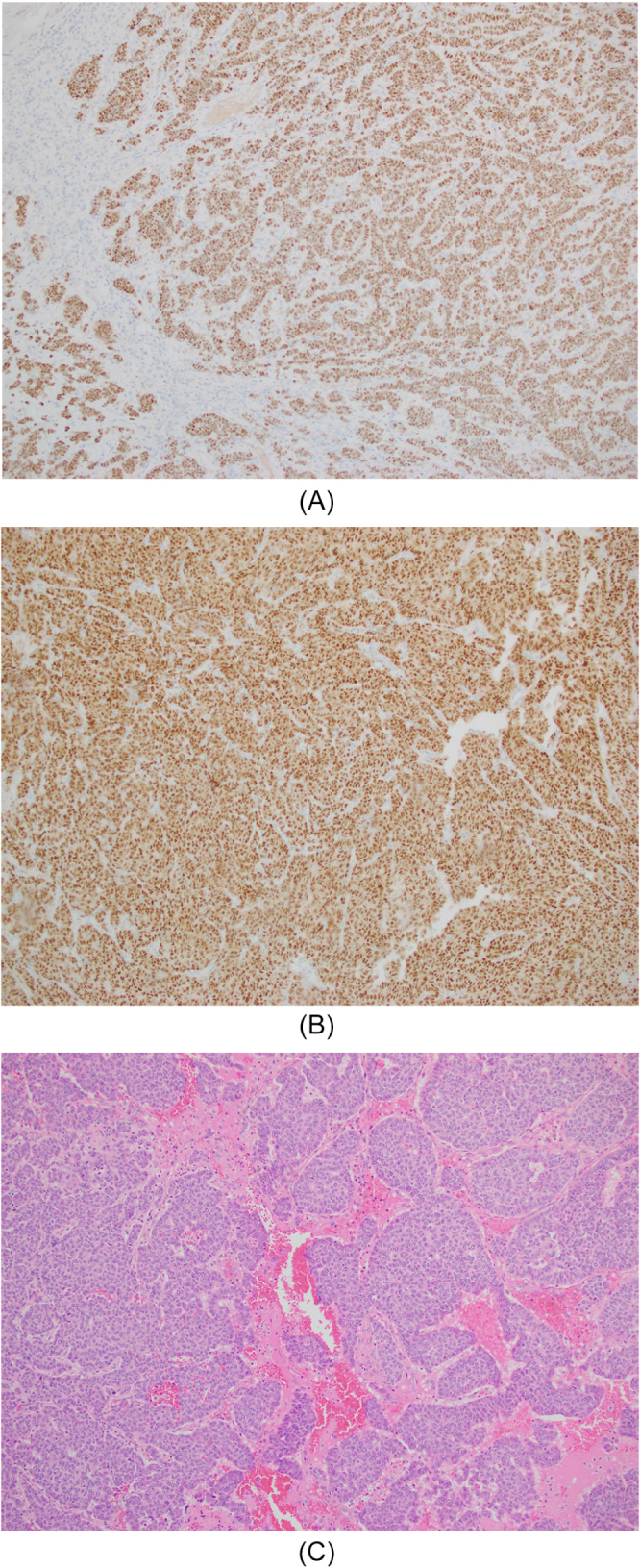
(A) estrogen staining showed moderately positive ER (clone SP-1) in >95% of cells. (B) GATA-3 immunohistochemistry with strongly positive nuclear staining. (C) Hematoxylin and eosin stain showed metastatic breast adenocarcinoma.

Immunohistochemistry of the tissue (Fig. 3a-c) revealed moderate estrogen receptor positivity (ER +), strong progesterone receptor positivity (PR >95%), low expression (1 +) of human epidermal growth receptor 2 (HER2), and a high Antigen Kiel 67 (Ki-67) index (60–70%), consistent with moderately differentiated metastatic adenocarcinoma of the adrenal gland with diffuse lymphovascular invasion. Tumor markers showed elevated cancer antigen (CA) 15-3 levels at 199 U/ml (normal: 0–31 U/ml) and CA27.29 at 238.1 U/ml (normal: ≤39.0 U/ml).

After more than a decade of remission, she was officially diagnosed with stage IV estrogen receptor/progesterone receptor (ER/PR) positive HER2 negative right breast cancer. Positron Emission Tomography (PET) scan results were unremarkable except for the 2.6 × 1.9 cm right adrenal nodule that has a maximum standardized uptake value (SUV_max_) of 4.8. The mass is indeterminate and could be a functional adrenal adenoma. Endocrine therapy was initiated with exemestane, a steroidal aromatase inhibitor, and follow-up appointments are scheduled to monitor for any additional metastases.

## Clinical discussion

Breast cancer is one of the most common cancers in women in the United States, with its median age of diagnosis being around 62 years of age^[7]^. Metastases of this cancer are most common in bone tissue, in the liver and in the lungs^[8]^. Metastasis to the adrenal glands in breast cancer is typically associated with aggressive systemic metastases and is found in 58% of autopsies with metastatic breast cancers patients^[9]^. However, isolated adrenal metastasis of breast cancer is very rare, so adrenalectomy for breast cancer metastasis is rarely performed^[9]^. Analysis of breast cancer metastatic pattern has shown that lobular tumors, as opposed to ductal tumors, were more likely to metastasize to the adrenal glands (0.6% vs 0%)^[10]^.

On the other hand, the adrenal gland is a relatively frequent site of metastasis due to its rich sinusoidal blood supply^[11,12]^. In fact, the most common tumors in the adrenal gland are metastatic tumors. This most often occur from lung, renal, and gastrointestinal cancer^[13]^, with squamous cell carcinoma and adenocarcinoma being the most commonly encountered histological diagnoses. In contrast, adrenal metastases from breast cancer account for only 2.9% of all adrenal metastases and are most often associated with synchronous metastases at other extra-adrenal sites^[14]^.

The median time from breast cancer to initial adrenal metastasis is 1–4 years^[15]^. This unusual case, however, highlights the indolent yet persistent nature of hormone receptor-positive breast cancer, and to our knowledge, is the first case with metastasis presenting over a decade postdiagnosis. A previous study performed with 1702 women with ER +/HER2- breast cancer, similar to our patient, showed that prognostic EndoPredict (EP) score- a multigene score that combines the expression levels of proliferative and ESR1 signalling/differentiation-associated genes, identifies late relapse events^[16]^. Moreover, clinical factors such as increased tumor size and nodal positivity have also been shown to be associated with late relapse^[17]^.

Table 1 summarizes other case reports we have found on metachronous solitary adrenal metastases from breast cancer, their presentation, years after diagnoses of breast cancer and treatment performed.

**Table 1:** Summary of published case reports on metachronous solitary adrenal metastases from breast cancer. Notably, all previously reported cases had a latency period of less than 10 years.

Literature	Breast cancer diagnosis	Interval between primary treatment and adrenal metastasis	Presenting complaint of adrenal metastasis	Treatment
Mizuyama *et al*^[18]^	HER-2 enriched breast cancer	2 years	Asymptomatic; found on abdominal computed tomography	Laparoscopic adrenalectomy; 12 cycles of chemotherapy with paclitaxel and trastuzumab, followed by trastuzumab monotherapy
Andjelić-Dekić *et al*^[19]^	Right invasive ductal breast carcinoma; Stage IIIA (T2N2M0)	3 years	Asymptomatic; found on abdominal computed tomography	Left adrenalectomy; 6 cycles of docetaxel with trastuzumab
Demirci *et al*^[20]^	Mucinous breast carcinoma	4 years	N/A	Laparoscopic adrenalectomy
Liu *et* *al*^[21]^	Invasive ductal carcinoma	2 years	Asymptomatic; found on abdominal CT	Left adrenalectomy
He *et* *al*^[22]^	Invasive ductal carcinoma	5 years	Asymptomatic; found on abdominal CT	Left adrenalectomy
Akhtar *et al* 2012^[23]^	Invasive ductal carcinoma	1 year	Pain in abdomen and shortness of breath	Left adrenalectomy
Nakagawa *et al*^[9]^	Invasive ductal carcinoma	9 years	Asymptomatic; found on abdominal CT	Endoscopic adrenalectomy
Sun et al^[24]^	Invasive mucinous carcinoma	5 years	Asymptomatic; found on abdominal CT	Right adrenalectomy
Nuno André Barros *et al*^[25]^	Invasive ductal carcinoma	5 years	Right hypochondriac pain	Total right adrenalectomy

Notably, all previously reported cases had a latency period of less than 10 years (mean duration: 4 years). Moreover, most of the cases were incidentally diagnosed on CT, with only a few presenting with abdominal pain. All the reported cases were treated with laparoscopic (most common), open, or endoscopic adrenalectomy. Some of the cases were additionally treated with chemotherapy.

There are no definitive guidelines for the treatment of adrenal metastasis from breast cancer^[15]^. In a previous single-institution study, adrenal metastasectomy performed with curative intent resulted in median survival of 30 months and 5-year survival of 31%. Shorter survival was associated with primary lung cancer, a short disease-free interval, and synchronous metastases^[13]^. Another study performed in 2021 suggested that aggressive resection of isolated adrenal metastases from breast cancer associated with adjuvant treatment allows excellent disease-free survival with minimal surgical morbidity and no surgery-related mortality^[14]^. Although surgical management is often the first choice, emerging therapies such as ablation are increasingly used, particularly for oligometastatic adrenal tumors due to their association with fewer postoperative complications. It is important to note, however, that ablation is found to be inferior to surgical excision in cases of isolated adrenal metastasis, such as in our case, according to a retrospective cohort conducted in 2021^[18]^. Other alternative treatment modalities include radiotherapy^[19,20]^ and thermotherapy^[26]^, which have been used in the past and have shown variable outcomes. However, they carry potential risks including hemodynamic instability and adrenal insufficiency.

Overall, this case documents an exceptionally delayed presentation of solitary adrenal metastases from breast cancer, occurring 15 years after the primary diagnosis, and presenting solely with hypercalcemia. It emphasizes the importance of considering metastases in the differential diagnoses of breast cancer patients in remission, regardless of the duration of said remission. In addition, our case supports existing evidence that adrenalectomy for solitary adrenal metastasis can provide clinical benefits in selected patients.

### Strengths and limitations

The strength of this report lies in its novelty and relevancy to clinicians, illustrating the importance of a multidisciplinary tumor board involvement and the feasibility of surgical resection in selected cases. However, as a single case, generalizability is limited, and long-term outcome data are still pending.

## Data Availability

Data sharing is not applicable to this article.

## Research registration unique identifying number (UIN)

This is a case report and hence, registration was not done.
